# Genome skimming reveals the complete chloroplast genome of *Ampelocalamus naibunensis* (Poaceae: Bambusoideae: Arundinarieae) with phylogenomic implication

**DOI:** 10.1080/23802359.2016.1214550

**Published:** 2016-09-04

**Authors:** Xian-Zhi Zhang, Si-Yun Chen

**Affiliations:** a College of Forestry, Northwest A&F University, Yangling, Shaanxi, China;; b Germplasm Bank of Wild Species, Kunming Institute of Botany, Chinese Academy of Sciences, Kunming, Yunnan, China

**Keywords:** Chloroplast genome, *Ampelocalamus naibunensis*, genome skimming, phylogenomics

## Abstract

*Ampelocalamus naibunensis* is one drooping bamboo with an important ornamental value endemic to Taiwan Island. To date, the genetic and genomic information of this species is little known. Here we characterized the complete chloroplast genome of *A. naibunensis* using genome skimming approach. The complete chloroplast genome is 139,860 bp, with a large single copy region (LSC) of 83,380 bp and a small single copy region (SSC) of 13,014 bp separated by a pair of inverted repeats (IRs) of 21,822 bp. The genome encodes a total of 129 genes, of which 111 are unique, containing 76 protein-coding genes, 4 ribosomal RNAs and 31 transfer RNAs. Sixteen distinct genes contain one or two introns, and the GC content of the cp genome is 38.9%. Phylogenomic analysis strongly supports the placement of *A. naibunensis* in the *Chimonocalamus* lineage (III), distantly related to *A. calcareus* (XI) within temperate woody bamboos.

*Ampelocalamus naibunensis* (Hayata) T.H. Wen is stenochoric and endemic to southern Taiwan Island, with important ornamental value for its apically drooping appearances (Li et al. 2006). This species plays a crucial role in understanding the intriguing biogeographic pattern of *Ampelocalamus*, an obviously discontinuous distribution of mainland China-Taiwan Island-Hainan Island (Wu et al. 2010; Zhang et al. 2016a), as well as the spatial and temporal diversification of temperate woody bamboos (Arundinarieae) in Bambusoideae (Zhang et al. 2016b). In this study, we assembled and characterized the complete chloroplast (cp) genome of *A. naibunensis* via genome skimming approach (Straub et al. 2012), which will provide additional temp-spatially evolutionary information of the special forestry-adapted group of the grass family.

Total genomic DNA was extracted from fresh leaves of *A. naibunensis* grown in Kunming Botanic Garden in Kunming, Yunnan province of China. The voucher specimen was deposited at the Herbarium of Kunming Institute of Botany (accession number Zhang12318). Illumina paired-end (PE) library was constructed and sequenced (2 × 100 bp) from fragmented genomic DNA in Beijing Genomics Institute (BGI) in Shenzhen, China. The cp genome *de novo* assembly was performed using CLC Genomics Workbench v7.5 software (CLC Bio, Aarhus, Denmark). Obtained cp contigs (length >300 bp and sequence coverage >50) were retrieved and ordered by a BLAST search against the reference sequence of *Phyllostachys edulis* (HQ337796). The finished cp genome was annotated using DOGMA (Wyman et al. 2004), coupled with manual adjustments.

The assembled cp genome of *A. naibunensis* (GenBank accession KX372537) is 139,860 bp in size with high coverage (mean value 369.5), showing a typical quadripartite structure: two inverted repeats (IRs) of 21,822 bp separating one large single copy region (LSC) of 83,380 bp and one small single copy region (SSC) of 13,014 bp. A total of 129 genes are contained in the cp genome, of which 111 are unique, including 76 protein-coding genes, 4 ribosomal RNAs and 31 transfer RNAs. The overall GC content of the cp genome is 38.9%. One protein-coding gene (*ycf3*) contains two introns while another 9 genes (*atpF*, *ndhA*, *ndhB*, *petB*, *petD*, *rpl16*, *rpl2*, *rps12*, *rps16*) contain one intron each. Protein-coding regions contribute 42.4% of *A. naibunensis* cp genome which bears a high level of conservation in terms of gene content, gene order, GC content and the size of IRs compared with other already published cp genomes of Arundinarieae (Zhang et al. 2011).

Phylogenomic analysis was performed based on the complete cp genomes of 29 bamboos using RAxML v.8.2.8 (Stamatakis 2014). We found that *A. naibunensis* clustered in the *Chimonocalamus* clade (III), being sister to *C. longiusculus* with high-support value (Figure 1). *Ampelocalamus naibunensis* is distantly related to *A. calcareus*, the earliest divergent lineage (XI) of Arundinarieae (Zhang et al. 2016b). The phylogenetic relationships of the 11 major lineages (I to XI) of Arundinarieae recovered here is congruent with Ma et al. (2014). The short internodes connected by long branches in the ML tree suggested a probable recent rapid radiation of Arundinarieae (Zhang et al. 2016b).

**Figure 1.**
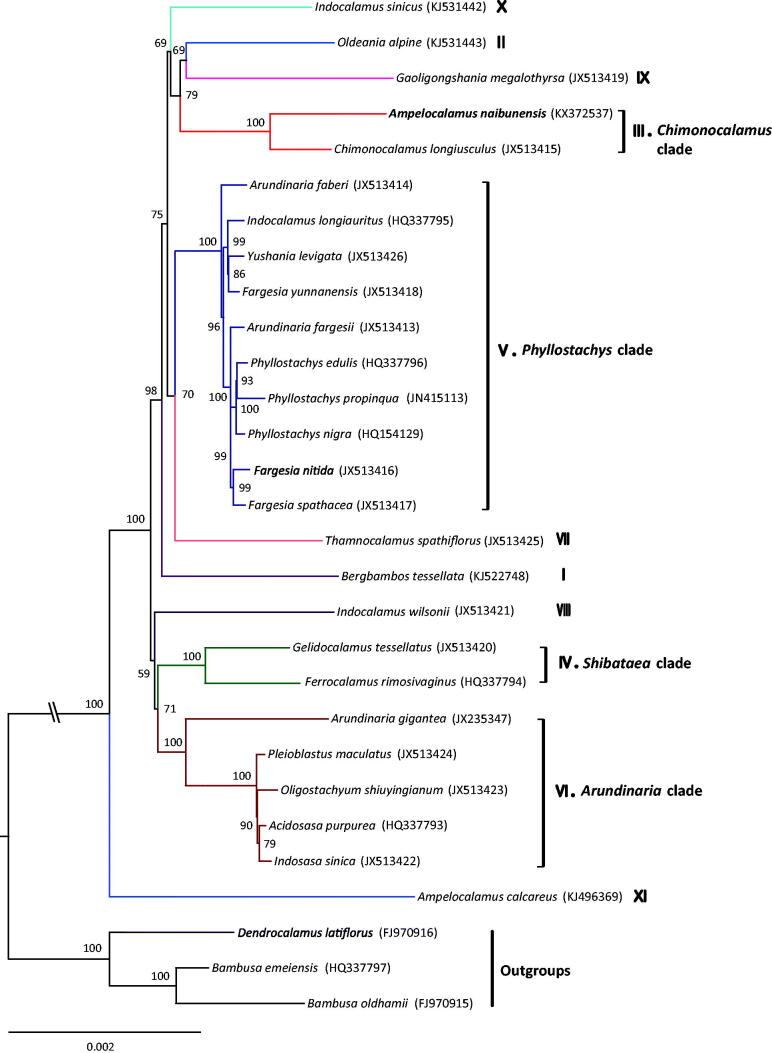
Maximum likelihood tree inferred from 29 woody bamboo chloroplast genomes. Colored branches indicate the 11 Arundinarieae lineages (I to XI). The position of *Ampelocalamus naibunensis* is shown in bold. Values associated with nodes are bootstrapping supports.
